# The glutathione S-transferase gene *LaGSTU6* promotes somatic embryogenesis in a recalcitrant lily genotype by regulating scales redox homeostasis

**DOI:** 10.1186/s43897-026-00244-9

**Published:** 2026-10-09

**Authors:** Yue Ma, Hongyu Li, Hongmei Sun

**Affiliations:** 1 Key Laboratory of Protected Horticulture of Education Ministry, College of Horticulture, Shenyang Agricultural University, Shenyang, 110866 China; 2 National and Local Joint Engineering Research Center of Northern Horticultural Facilities Design and Application Technology, Shenyang, 110866 China; 3 College of Life Science and Bioengineering, Shenyang University, Shenyang, People’s Republic of China

**Keywords:** *Lilium amabile* Palib., Somatic embryogenesis, Genotype dependent, Glutathione, *GST*, Cellular redox homeostasis

## Abstract

Genotypic recalcitrance limits efficient somatic embryogenesis (SE). In this study, an SE induction system for the recalcitrant species *Lilium amabile* Palib. was established by modulating redox homeostasis. The first SE in this species was achieved by supplementing MS medium containing 1.0 mg·L^−1^ picloram (PIC) with 150.0 mg·L^−1^ glutathione (GSH), which increased SE induction rate from 0 to 33.3%. Physiological analysis revealed that GSH treatment upregulated endogenous GSH and key antioxidant enzymes, glutathione peroxidase (GPX) and glutathione S-transferase (GST). Specifically, their activities were elevated 1.9- and 9.8-fold higher, respectively, than those of control. This synergistic action scavenged hydrogen peroxide (H₂O₂), thereby alleviating oxidative stress. Furthermore, *LaGSTU6* was identified as a core regulator of SE. In the SE-efficient lily *Lilium martagon* var. *pilosiusculum* Freyn, transient silencing of *LaGSTU6* delayed embryogenesis initiation, caused severe browning, induced H₂O₂ accumulation, and reduced the antioxidant capacity, yielding a significant decrease in both the SE induction rate and efficiency. These findings preliminarily confirm that *LaGSTU6* is a critical regulator of SE competence in lilies. Thus, we overcame genotypic recalcitrance and established the first SE induction system in *Lilium amabile* Palib. Our findings provide insights into the study of totipotency and inform strategies for regenerating recalcitrant germplasms.

**Supplementary Information:**

The online version contains supplementary material available at https://doi.org/10.1186/s43897-026-00244-9.

## Core

Somatic embryogenesis (SE) in the recalcitrant species *Lilium amabile* Palib. was achieved by adding GSH to the culture medium, which elevated the SE induction rate from 0 to 33.3%. This treatment enhanced antioxidant enzyme activities and reduced H₂O₂ accumulation. *LaGSTU6* was identified as a key regulator, as its silencing suppressed SE competence. This study provides an efficient SE system and new insights into plant totipotency.

## Gene and accession numbers

Gene information used in this study is available from Liliales Genome Database (https://lgd.njau.edu.cn) under the following accession number: *LaGSTU6* (Lily02G48630); *LaGSTU1* (Lily11G50430); *LaGSTU17* (Lily04G32710); *LaGST23-1* (LregChr09G036890); *LaGST23-2* (lili00G317820.t1); *LaGSTT1* (LregChr10G032570); *LaGSTX* (lili00G408950.t1); *LaIAA1* (Lily11G24240); *LaIAA1-1* (lili00G256890.t1); *LaIAA1-2* (LregChr12G031990); *LaIAA10* (LregChr04G033770); *LaIAA13* (lili00G099330.t1); *LaIAA30* (Lily04G31370); *LaIAA33* (lili00G035080.t1); *LaARF9* (LregChr06G045980); *LaARF15-1* (Lily01G47960); *LaARF15-2* (LregChr01G005210); *LaARF19-1* (Lily08G65450); *LaARF19-2* (LregChr06G000610).

## Introductions

Somatic embryogenesis (SE), which constitutes an important manifestation of in vitro cellular reprogramming, serves as a versatile platform for critical applications in plant biotechnology, such as clonal propagation, genetic engineering, rapid multiplication, and trait-specific improvement (Zimmerman 1993; Youngstrom et al. 2014; Mao et al. 2019; Ramírez-Mosqueda 2022; Li et al. 2025; Moshood et al. 2025). However, its application is restricted by the existence of pervasive genotype dependency, with the regeneration capacity varying significantly even among closely related varieties (Wang et al. 2021; Andrade et al. 2024). For example, in *Hevea brasiliensis*, only a limited number of genotypes can efficiently establish high-quality clonal plants via SE (Wang et al. 1980; Blanc et al. 2002; Hua et al. 2010), while most elite germplasms fail to provide a stable regeneration system, severely impeding industry advancement (Wang et al. 2023). Similarly, genotype-dependent regeneration obstacles are notable in *Gossypium hirsutum*, thus significantly delaying the widespread adoption of gene-edited elite varieties (Khan et al. 2010; Zhang et al. 2011).

Recent breakthroughs in regeneration biology have created new avenues to overcome genotypic constraints. The regeneration factor REF1 was demonstrated to significantly increase the regeneration efficiency in recalcitrant species such as *Solanum lycopersicum*, *Glycine max*, and *Zea mays* through the PORK-WIND1 pathway (Yang et al. 2024). Furthermore, the expression of peptide signaling molecules such as phytosulfokine (PSK) has been reported to promote SE in *Nicotiana benthamiana* (Joo et al. 2022), *Brassica napus* (Mubi et al. 2024), and *Cunninghamia lanceolata* (Hao et al. 2023), which suggests the importance of molecular approaches for optimizing SE induction. However, the genotype dependence of SE is highly species specific, and universal solutions have not been established to date.

*Lilium amabile* Palib., which represents an important wild lily germplasm resource in China, exhibits both drought tolerance and shade adaptability (Xu and Lei 2010). However, owing to habitat degradation and anthropogenic disturbance, its wild populations continue to decline, and natural population recovery remains ineffective. SE, which is recognized as an ideal system for achieving high-efficiency plant regeneration and genetic improvement owing to its high propagation rate and genetic stability (Yan et al. 2020a, b), has been successfully established in several lily species, including *Lilium pumilum* DC. Fisch. (Yan et al. 2022; Sun et al. 2023; Wang et al. 2024). Nevertheless, existing SE induction systems offer limited effectiveness for *Lilium amabile* Palib., and a reliable SE protocol has yet to be established, significantly limiting efforts toward wild population restoration and germplasm use. Therefore, the development of an efficient SE system for *Lilium amabile* Palib. has become an urgent task to advance its conservation and sustainable use.

Glutathione S-transferases (GSTs), which are encoded by multigene families, constitute a superfamily of enzymes that primarily catalyze the conjugation of GSH with electrophilic substrates. The enzymes play crucial roles in shaping cellular detoxification, oxidative stress responses, and reactive oxygen species (ROS) metabolism (Noctor et al. 2024). The results of functional studies have indicated that the loss of GST activity can cause severe redox disruption. For example, in *Cucumis melo*, disruption of *CmGSTU25* leads to ROS accumulation and aggravated membrane lipid peroxidation (Xu et al. 2024). Furthermore, *GSTs* have been reported to be specifically expressed in genotypes with high embryogenic capacity, such as in *Cyclamen persicum* and *Cichorium hybrid*, suggesting their potential involvement in SE regulation (Galland et al. 2007; Hoenemann et al. 2010). Nevertheless, the specific mechanistic role of *GSTs* in reprogramming cell fate and modulating SE competence in lilies remains unclear.

To elucidate the genotypic dependency of SE in lilies, an SE-efficient genotype (EG), i.e., *Lilium martagon* var. *pilosiusculum* Freyn, was compared with a recalcitrant genotype (RG), i.e., *Lilium amabile* Palib., in this study. Untargeted metabolomics revealed that GSH metabolism was the most significantly altered pathway. Exogenous GSH application effectively alleviated the regeneration barrier in the RG, thereby increasing its SE induction rate from 0 to 33.3%. Physiologically, GSH treatment increased endogenous GSH levels, enhanced the activities of GPX and GST, and reduced H₂O₂ accumulation, thereby mitigating oxidative stress. Furthermore, *LaGSTU6* was identified as a core regulator of SE. The transient silencing of *LaGSTU6* in the EG caused delayed embryogenic initiation, exacerbated browning, increased H₂O₂ accumulation, and decreased the antioxidant capacity, ultimately resulting in a significant decrease in the SE induction rate and efficiency. These findings preliminarily confirm that *LaGSTU6* is a pivotal regulator of SE competence in lilies. Our study establishes the first redox-based SE system for *Lilium amabile* Palib., thereby providing a strategy for overcoming regeneration barriers and advancing germplasm innovation.

## Results

### Genotypic differences in SE competence in lilies

**Fig. 1:**
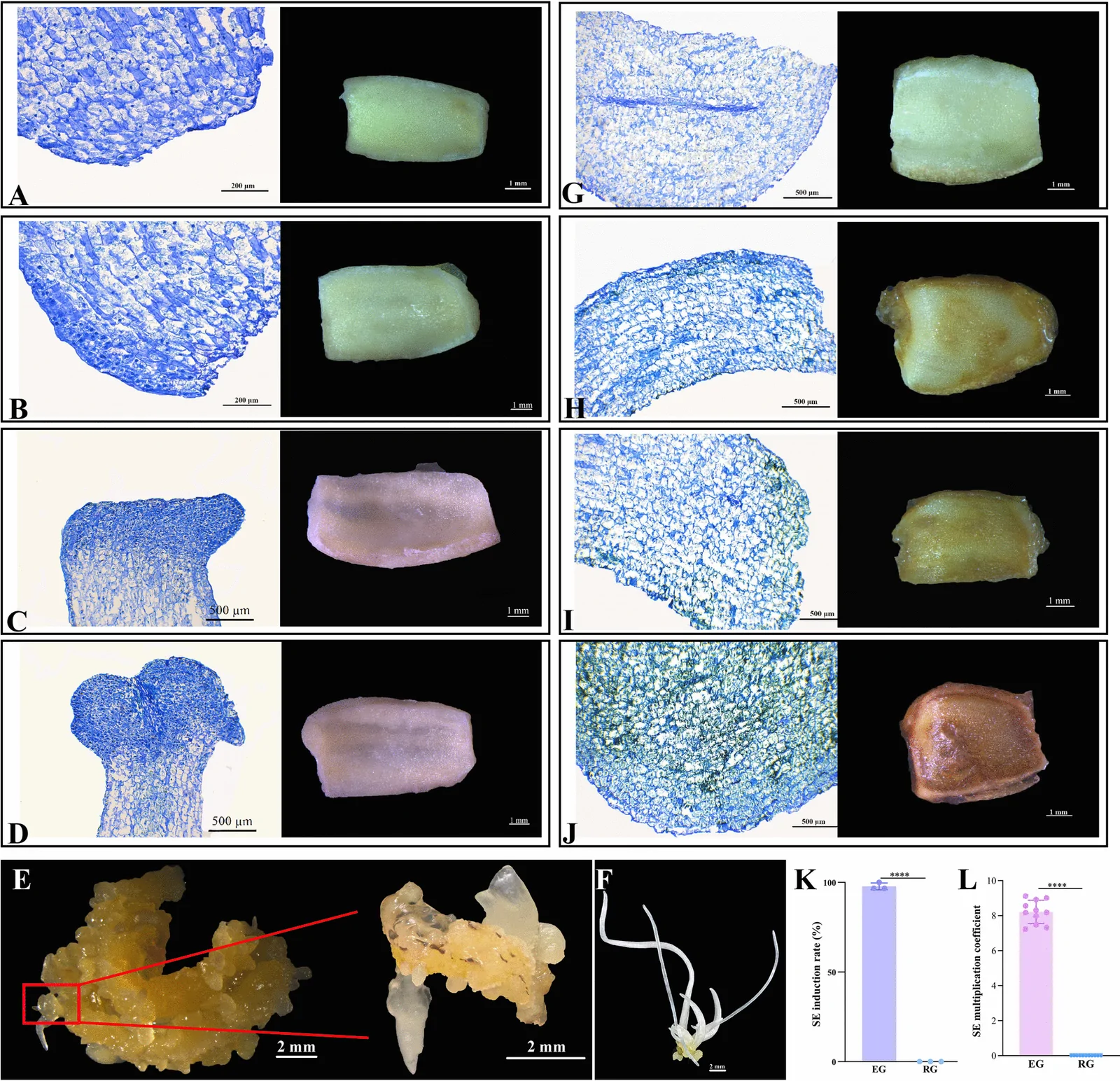
Genotypic differences in the somatic embryogenesis (SE) capacity of lilies. **A**–**D‌** *Lilium martagon* var. *pilosiusculum* Freyn (efficient genotype, EG) scales at 0, 6, 9, and 12 d after induction. **E** 60-day-induced somatic embryos of the EG. **F** Regenerated plantlet from somatic embryo germination. **G**–**J** *Lilium amabile* Palib. (recalcitrant genotype, RG) scales induced for 0, 6, 9, and 12 d. Notes‌: All the scales were cultured on MS medium supplemented with 1.0 mg·L.^−1^ picloram (PIC). **K** SE induction rate comparison between the EG and RG. Data are presented as the mean ± standard deviation (SD) from three independent biological experiments (*n* = 3), each performed with 30 scales analyzed per replicate. **L** SE induction coefficient comparison between the EG and RG. Data are presented as the mean ± SD from three independent experiments, each performed with eleven biological replicates. Data are presented as the mean ± SD from eleven independent experiments (*n* = 11), each performed with three biological replicates. Statistical significance was determined via Student’s *t* test (****, *p* < 0.0001)

We compared two lily genotypes with contrasting SE capacities, namely, *Lilium martagon* var. *pilosiusculum* Freyn (EG) and *Lilium amabile* Palib. (RG). The SE induction rate of the EG was 100%, and the induction coefficient reached 9.0 on induction medium (Fig. 1K, L), whereas the RG failed to generate SE under any tested condition and exhibited severe explant browning (Table S2). Histomorphological observations revealed distinct differences during early induction. In the EG, active division of epidermal cells was observed at 6 d after induction, with yellowish protuberances emerging at 12 d. These protuberances eventually formed embryogenic calluses and regenerating plantlets (Fig. 1A–F). In contrast, the RG exhibited browning from the margins toward the center at 6 d, which was accompanied by characteristics of programmed cell death. With prolonged culture, the level of browning increased without the formation of embryogenic structures (Fig. 1G–J). Thus, the period of 6 d after induction was identified as the critical stage for determining embryogenic cell initiation in the EG and browning in the RG. Although these two lilies share similar morphological traits, such as leaf shape and flower posture (Rong et al. 2011; Zhou et al. 2018), which suggests a close genetic relationship, their SE capacities differ significantly. Therefore, in the subsequent investigations, the EG and RG were employed to study the genotype-dependent mechanisms underlying SE in lilies.

### Differences in metabolism during EG and RG induction

**Fig. 2:**
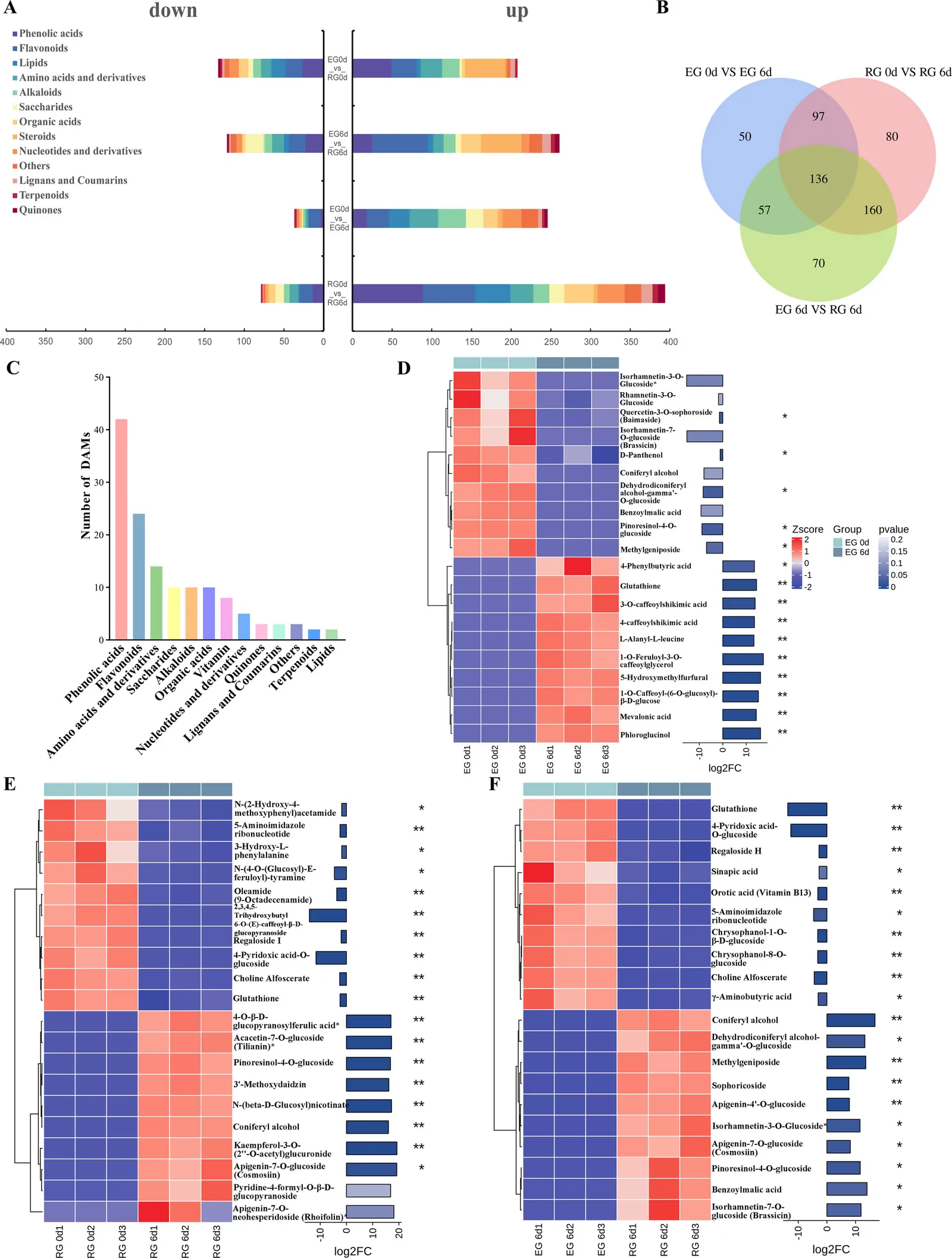
Identification of differentially accumulated metabolites (DAMs) between the EG and RG. **A** Categorization of upregulated and downregulated DAMs across four comparison groups: EG 0 d VS RG 0d‌, EG 6 d VS RG 6d‌, EG 0 d VS EG 6 d,‌ and RG 0 d VS RG 6d. **B** Venn diagram showing overlapping DAMs among three critical comparisons: EG 6 d VS RG 6 d, EG 0 d VS EG 6 d, and RG 0 d VS RG 6d. A core set of 136 DAMs was identified. **C** Functional classification of the 136 shared DAMs. **D** Heatmap clustering of the DAMs in the EG 0 d VS EG 6 d comparison group. **E** Heatmap clustering of the DAMs in the RG 0 d VS RG 6 d comparison group. **F** Heatmap clustering of the DAMs in the ‌EG 6 d VS RG 6 d comparison group. Statistical significance of the DAMs was determined via one-way analysis of variance (ANOVA) (*, *p* < 0.05; **, *p* < 0.01)

Since the 6-d induction period is critical for determining cell differentiation, widely targeted metabolomics analysis was conducted for EG and RG samples collected at 0 and 6 d after induction. The results of principal component analysis (PCA) demonstrated high reproducibility between biological replicates and reliable data quality (Fig. S1A, B). An analysis of differentially accumulated metabolites (DAMs) revealed consistently increased accumulation levels of steroids, phenolic acids, and flavonoids in the RG both before and after induction (Fig. 2A). Among these compounds, phenolics and flavonoids function as substrates for polyphenol oxidase and readily induce enzymatic browning (Dong et al. 2016), which conforms with the severe browning phenotype observed in RG scales. In contrast, the EG exhibited significant accumulation of amino acids and their derivatives, alkaloids, and sugars after induction, suggesting increased energy demands for active cell division.

To identify major metabolites that regulate SE competence during induction, we screened of DAMs under the induced conditions. We prioritized DAMs from RG 0 d VS RG 6 d, EG 0 d VS EG 6 d, and EG 6 d VS RG 6 d. This three-tiered screening resulted in the identification of 136 DAMs (Fig. 2B), comprising primarily phenolic acids (32.1%), flavonoids (28.3%), and amino acid derivatives (21.6%) (Fig. 2C). The results of trend analysis revealed that pme1086 (GSH) and mws0093 (coniferyl alcohol) were significantly altered in all the compared groups (Fig. 2D–F). In the comparison between the EG-6d and RG-6d groups, GSH was the most downregulated metabolite, whereas coniferyl alcohol was the most notably upregulated metabolite. Therefore, these two metabolites were selected as key regulatory substances for subsequent investigation into the differential SE capacity.

### Functional validation of GSH as a central regulator controlling SE competence in lilies

**Fig. 3:**
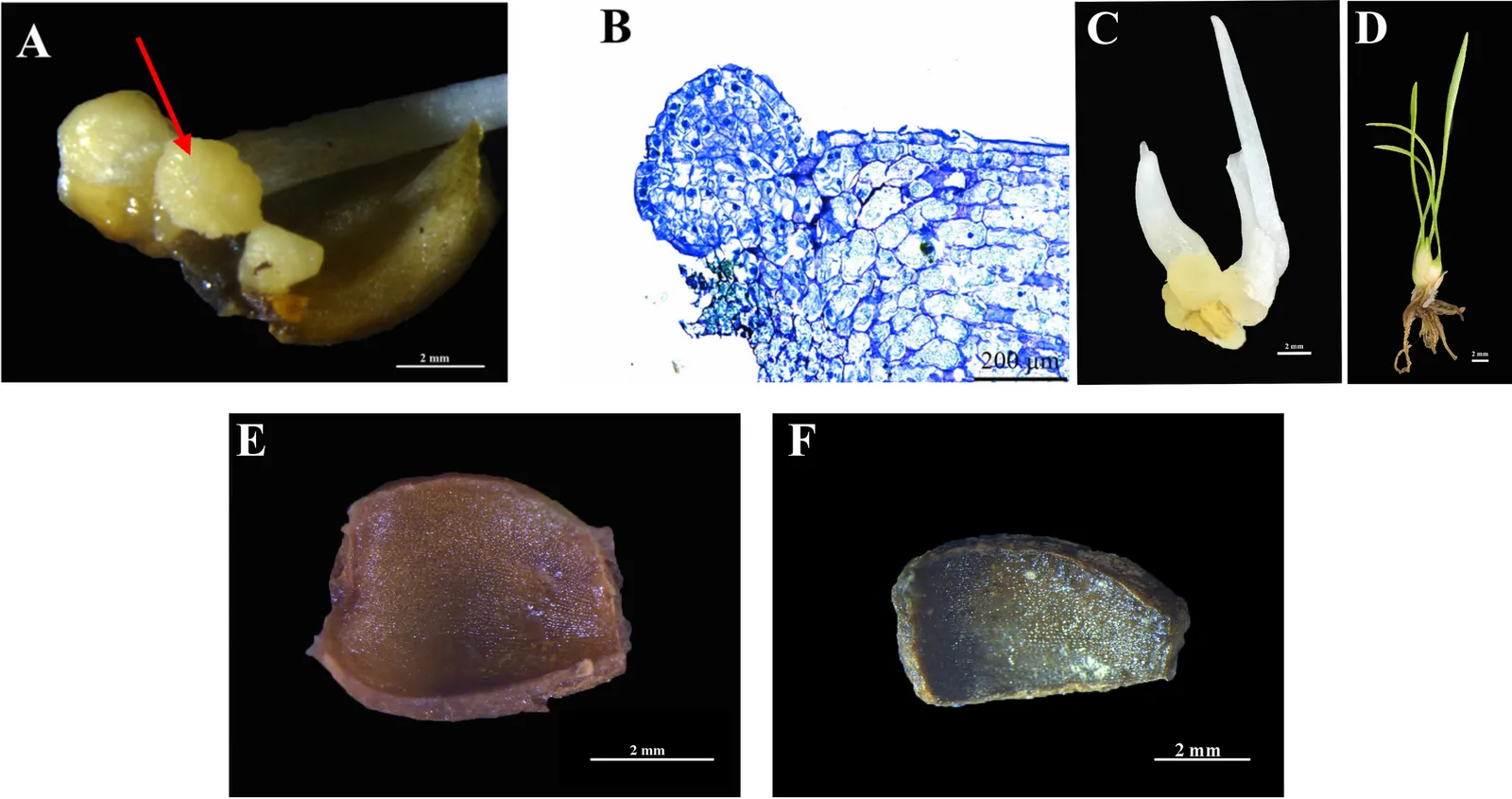
Histomorphological characterization of SE in the RG. **A** SE induction in RG scale segments cultured on induction medium supplemented with 150.0 mg·L^−1^ GSH for 30 d. The red arrows denote embryogenic calluses with distinct globular-stage embryos. **B** Histological analysis of RG tissues from (**A**). **C** Somatic embryos of the RG at the germination stage. **D** RG plantlet regenerated via SE. **E**, **F** RG scales cultured on MS medium supplemented with 150.0 mg·L^−1^ GSH (**E**) or 1.0 mg·L^−1^ PIC (**F**) for 30 d.

To evaluate the regulatory roles of the candidate metabolites, we treated RG and EG explants with exogenous GSH (0–300.0 mg·L^−1^) and lignin (a coniferyl alcohol polymer, 0–2.0 mg·L^−1^), respectively, in the induction medium. Remarkably, supplementation of the medium with 150.0 mg·L^−1^ GSH induced SE in the RG, with an induction rate of 33.3% (Fig. 3A, Table 1). The results of histological analysis confirmed active cell division in responsive RG tissues (Fig. 3B), and the resulting SE developed into complete plantlets (Fig. 3C, D). In contrast, the lignin treatment did not affect the SE frequency in the EG, which remained at 100%, although enhanced browning occurred at the wound sites (Fig. S2, Table 1). These data suggest that differential GSH levels may underlie the genotype-dependent SE ability and that GSH supplementation of the induction medium can restore SE competence in the RG.

**Table 1:** Effects of lignin and glutathione (GSH) on SE capacity in *Lilium*

Genotype	Metabolite	Concentration (mg·L^−1^)	SE induction rate (%)
EG	lignin	0.5	100.0 a
		1.0	100.0 a
		2.0	100.0 a
RG	GSH	75.0	0.0 d
		150.0	33.3 b
		300.0	20.0 c

Experiments were performed in triplicate with similar results. Statistical significance among groups was analyzed by one-way ANOVA with Tukey's multiple comparisons test. Different lowercase letters indicate statistically significant differences (*p* < 0.05)

To determine whether GSH alone is sufficient, we next evaluated its effect on RG explants without the auxin analog PIC. SE could not be induced in explants cultured with GSH alone (Fig. 3E), similar to those treated with PIC alone (Fig. 3F). However, the degree of tissue browning was significantly reduced in the GSH-only group compared with that in the PIC-only group. These results demonstrate that both GSH and PIC are necessary for achieving SE induction in the RG and likely function synergistically.

### Exogenous GSH promotes SE by restoring redox homeostasis

**Fig. 4:**
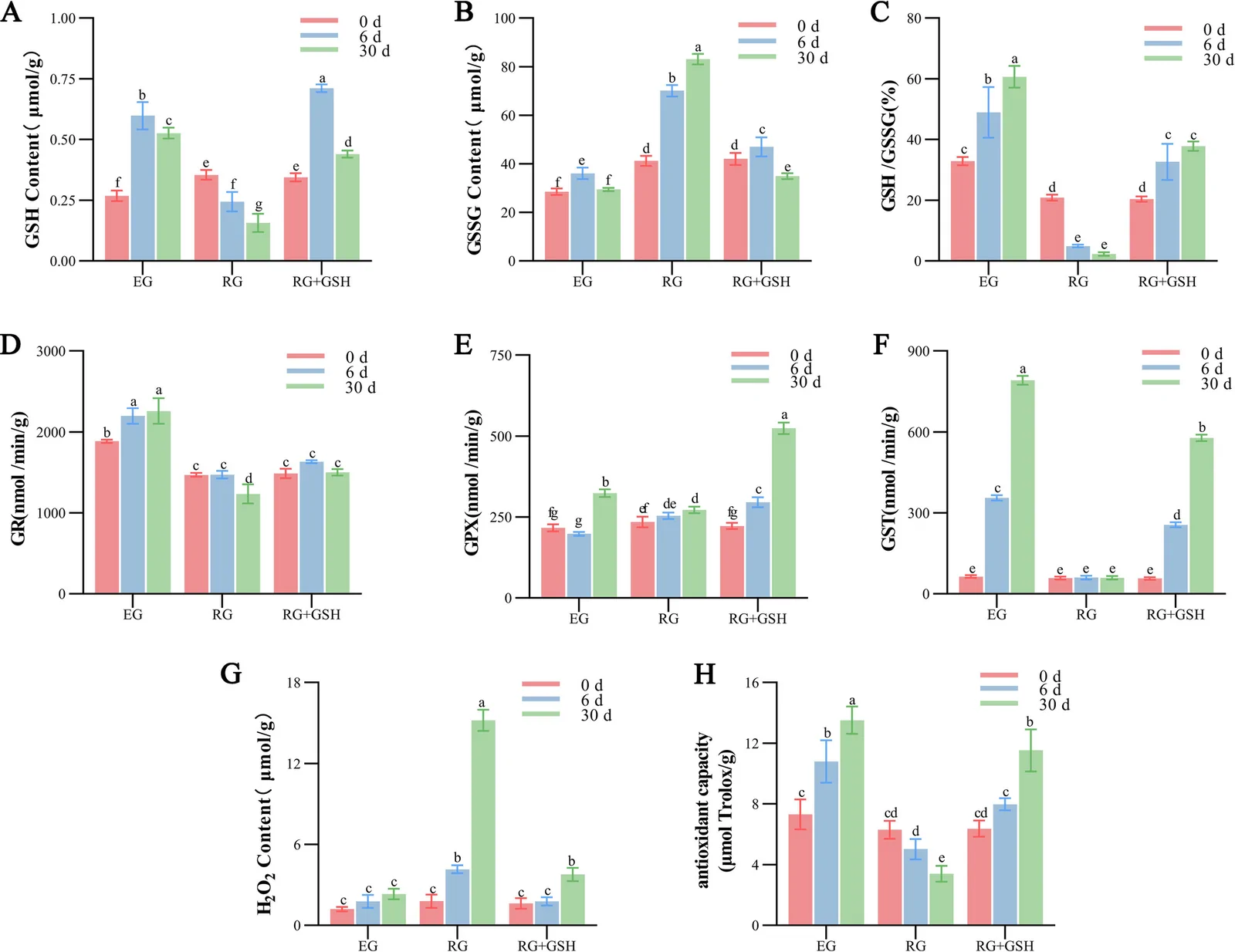
Exogenous GSH reprograms redox homeostasis to restore SE competency in the RG. **A**–**C** Temporal dynamics of the ‌endogenous GSH content (**A**), oxidized glutathione (GSSG) content (**B**), and GSH/GSSG ratio (**C**) in the EG, RG, and RG + GSH groups during SE induction (0, 6, and 30 d). **D**–**F** Antioxidant enzyme activities. (**D**) Glutathione reductase (GR) activity; (**E**) glutathione peroxidase (GPX) activity; (**F**) glutathione S-transferase (GST) activity. **G**–**H** Oxidative stress markers. (**G**) Hydrogen peroxide (H_2_O_2_) levels; (**H**) total antioxidant capacity. Data are presented as the mean ± SD from three independent experiments (*n* = 3), each performed with three biological replicates. Statistical significance was determined by one-way ANOVA followed by Tukey’s multiple comparisons test. The different lowercase letters indicate statistically significant differences (*p* < 0.05)

To elucidate the physiological mechanism through which GSH promotes SE in the RG, we analyzed the dynamics of GSH metabolism, key antioxidant enzyme activities, and oxidative stress markers at 0 d, 6 d (the critical period for embryogenic cell formation), and 30 d (the SE formation stage) after induction. The results revealed that in the RG, GSH was continuously consumed, and oxidized glutathione (GSSG) accumulated, indicating a state of oxidative stress. In contrast, the level of GSH in the EG peaked at 6 d, but a low GSSG content was maintained throughout induction. Exogenous GSH supplementation effectively reversed the redox imbalance in the RG, which increased endogenous GSH levels 2.0-fold relative to that in the RG control at 30 d and significantly reduced GSSG accumulation, thereby shifting the GSH/GSSG ratio toward a reduced state (Fig. 4A–C). Enzyme activity assays revealed that the GSH treatment notably increased GPX and GST activity levels in the RG by 1.9- and 9.8-fold, respectively, reaching or exceeding those in the EG (Fig. 4D–F). GPX is involved primarily in scavenging H₂O₂ and organic peroxides, whereas GST plays key roles in driving detoxification and antioxidant regulation. Their coordinated activation reshaped the antioxidant defense network. Furthermore, during induction, the RG exhibited substantial H₂O₂ accumulation and a continuous decrease in the total antioxidant capacity, thus reflecting severe oxidative stress. In contrast, the EG maintained lower H₂O₂ levels and a steadily increasing antioxidant capacity. Exogenous GSH supplementation significantly reduced the H₂O₂ content and increased the antioxidant capacity in the RG (Fig. 4G, H). These findings demonstrate that exogenous GSH supplementation increases endogenous GSH levels, coactivates GPX and GST, effectively reduces H₂O₂ accumulation, and reestablishes redox homeostasis conducive to SE initiation, thereby rescuing SE in the RG.

### Screening of key regulatory genes governing SE in the EG and RG

**Fig. 5:**
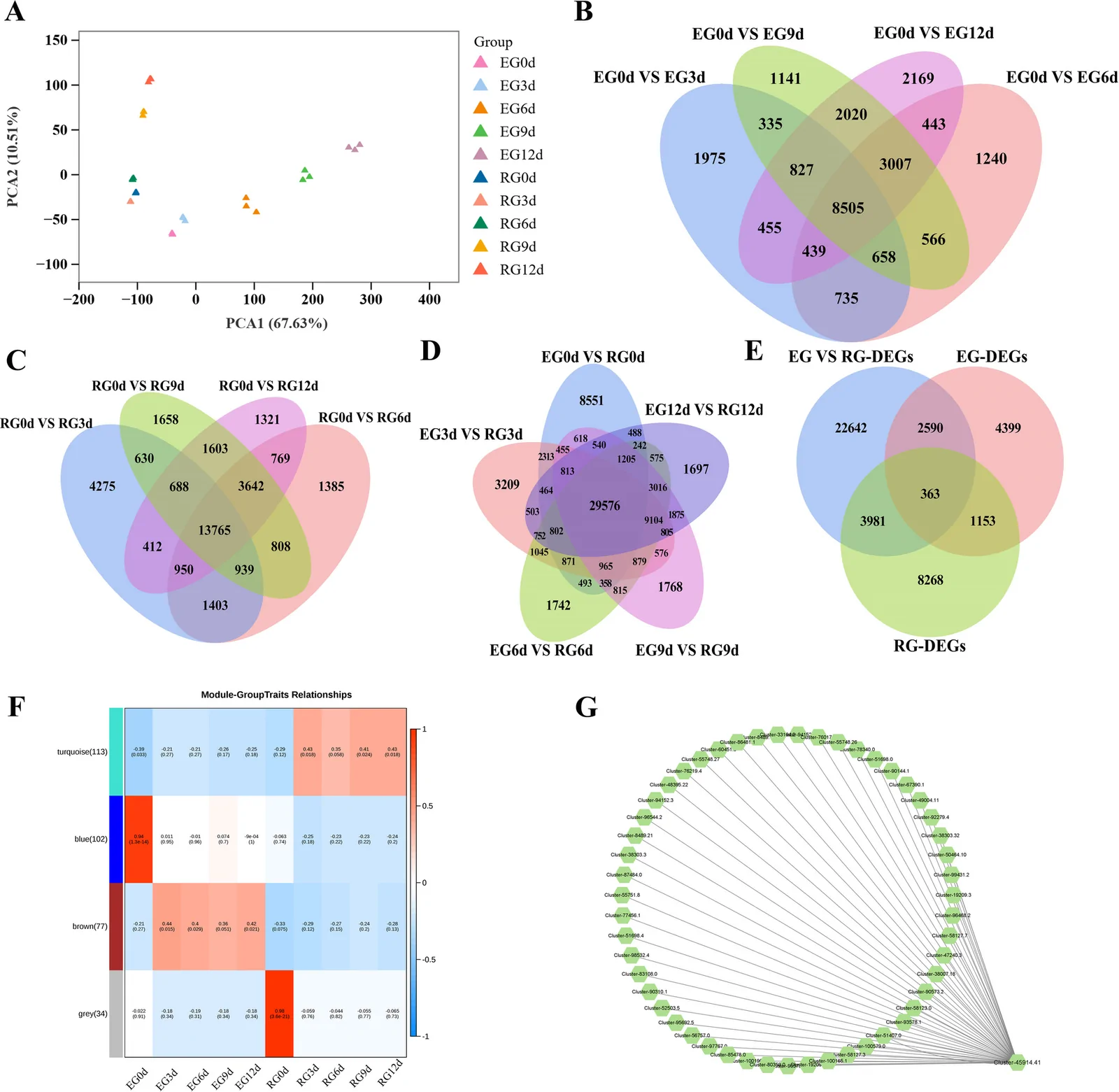
Transcriptome identification of differentially expressed genes (DEGs) between the EG and RG in SE. **A** Principal component analysis (PCA) of the RNA sequencing (RNA-seq) data from EG and RG explants at five induction time points (0, 3, 6, 9, and 12 d). **‌B** Venn diagram of DEGs identified in the EG (EG-DEGs) during early SE induction. **‌C** Venn diagram of RG-specific DEGs (RG-DEGs). **D** Genotype-specific DEGs (EG VS RG) at matched time points (0–12 d). **E** Overlap of DEG subsets from (**B**), (**C**), and (**D**). **F** Weighted gene coexpression network analysis (WGCNA) of the 363 common DEGs. **G** Coexpression network of hub genes within the brown module, visualized in Cytoscape

To elucidate the molecular mechanism through which GSH restored SE in the RG, we conducted RNA sequencing (RNA-seq) of both the EG and RG at five induction stages (0, 3, 6, 9, and 12 d). We obtained high-quality data, thereby assembling 198,950 unigenes with comprehensive functional annotations (Tables S3–S5). The PCA results revealed clear transcriptomic divergence between the EG and RG during induction (Fig. 5A). Differential expression analysis indicated three sets of differentially expressed genes (DEGs), namely, 8,505 DEGs during early SE induction in the EG (EG-DEGs, Fig. 5B), 13,765 DEGs in the RG (RG-DEGs, Fig. 5C), and 29,576 DEGs from pairwise comparisons between the EG and RG across all time points (EG VS RG-DEGs, Fig. 5D). A total of 363 common DEGs were identified from the intersection of these sets (Fig. 5E). Kyoto Encyclopedia of Genes and Genomes (KEGG) enrichment analysis revealed that GSH metabolism and phenylpropanoid biosynthesis were significantly amplified across all the comparison groups (Fig. S3). The expression patterns of selected key genes validated the reliability of the transcriptomic data (Fig. S4). Weighted gene coexpression network analysis (WGCNA) revealed a brown module strongly associated with SE competence in the EG, containing 77 genes (Fig. 5F). Coexpression network analysis revealed that the hub gene *LaGSTU6* (Cluster-45914.41) was highly connected (edge weight ≥ 0.8) to multiple functional genes, including six GST genes, twelve auxin-responsive transcription factors (AUX/IAA and B3-ARF), six LOB domain proteins involved in lateral root development, three genes associated with mitosis in cell cycle regulation (CPP and SNF2), and one calcium signaling gene (Fig. 5G, Table S6). Collectively, our findings suggest a synergistic interaction among GSH metabolism, auxin signaling, and calcium signaling in the acquisition of embryogenic competence. We propose that these pathways constitute an intrinsic regulatory network during SE. The core function of this network is to integrate redox signals with hormone and calcium cues, thereby coordinately regulating cellular redox homeostasis and reprogramming to precisely control the acquisition of embryogenic competence. This model establishes a molecular framework for investigating the synergistic mechanism between GSH and auxin.

The obtained findings indicate that during SE in the RG, these pathways may constitute an intrinsic and coordinated regulatory network. We propose that the core function of this network is to integrate redox signaling with hormone and calcium signaling, thereby jointly regulating cellular redox homeostasis and reprogramming processes. This integration precisely controls the acquisition of embryogenic competence, thereby establishing a specific molecular framework for further investigations into the synergistic mechanisms between GSH and auxin.

### Integrated transcriptomic and metabolomic analysis of the EG and RG

**Fig. 6:**
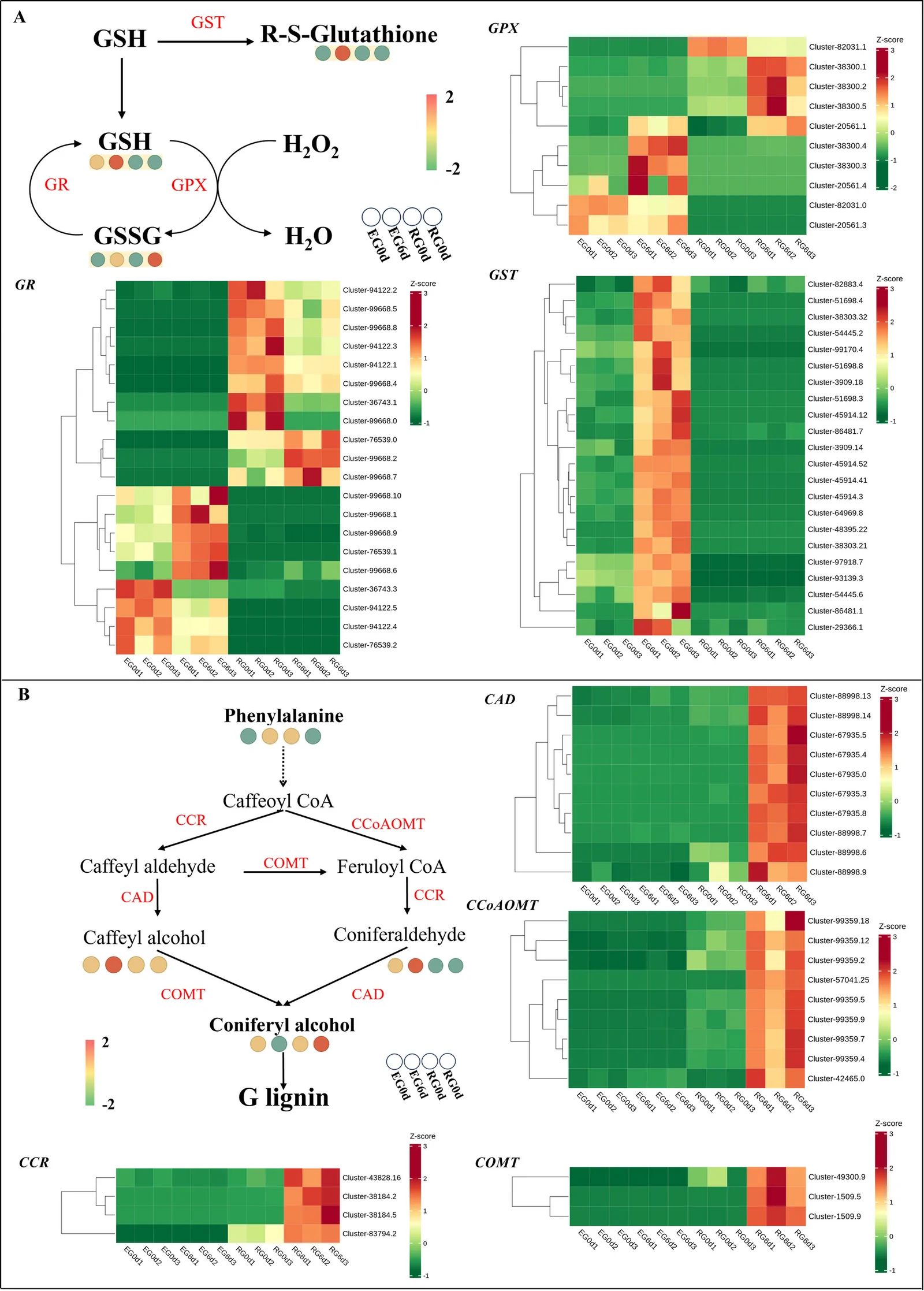
Coregulatory networks integrating transcriptomic and metabolomic data. **A** GSH metabolism pathway. **B** Phenylpropanoid biosynthesis pathway. These networks depict interactions between DEGs and DAMs

To systematically elucidate the key pathways regulating the differential SE capacity in lilies, we performed KEGG enrichment analysis for all the DAMs and DEGs. Consistent with our previous screening results, GSH metabolism and phenylpropanoid biosynthesis were significantly amplified in both the EG and RG (Fig. S5), highlighting the central role of these pathways in driving SE regulation. To elucidate their regulatory patterns, we mapped the core DAMs and DEGs from these two pathways (Fig. 6). Within the GSH metabolism pathway, the key metabolite GSH and its downstream core catalytic GSTs were observed at high levels in the EG, indicating that increased GSH metabolism and GST-mediated antioxidant activity underlie the high SE potential of the EG. In contrast, within the phenylpropanoid biosynthesis pathway, genes associated with lignin synthesis were significantly upregulated in the RG, suggesting that lignin accumulation during induction may constrain cellular developmental plasticity. In summary, the results of integrated analysis not only validated the key pathways identified previously but also revealed distinct metabolic processes in the two genotypes, namely, the GSH-mediated antioxidant system in the EG is activated, while lignin biosynthesis in the RG is overactivated. The coordinated differences observed at the metabolic and transcriptional levels provide a systematic mechanistic framework for understanding the difference in SE competence between the EG and RG.

### *LaGSTU6* positively regulates SE in lily

**Fig. 7:**
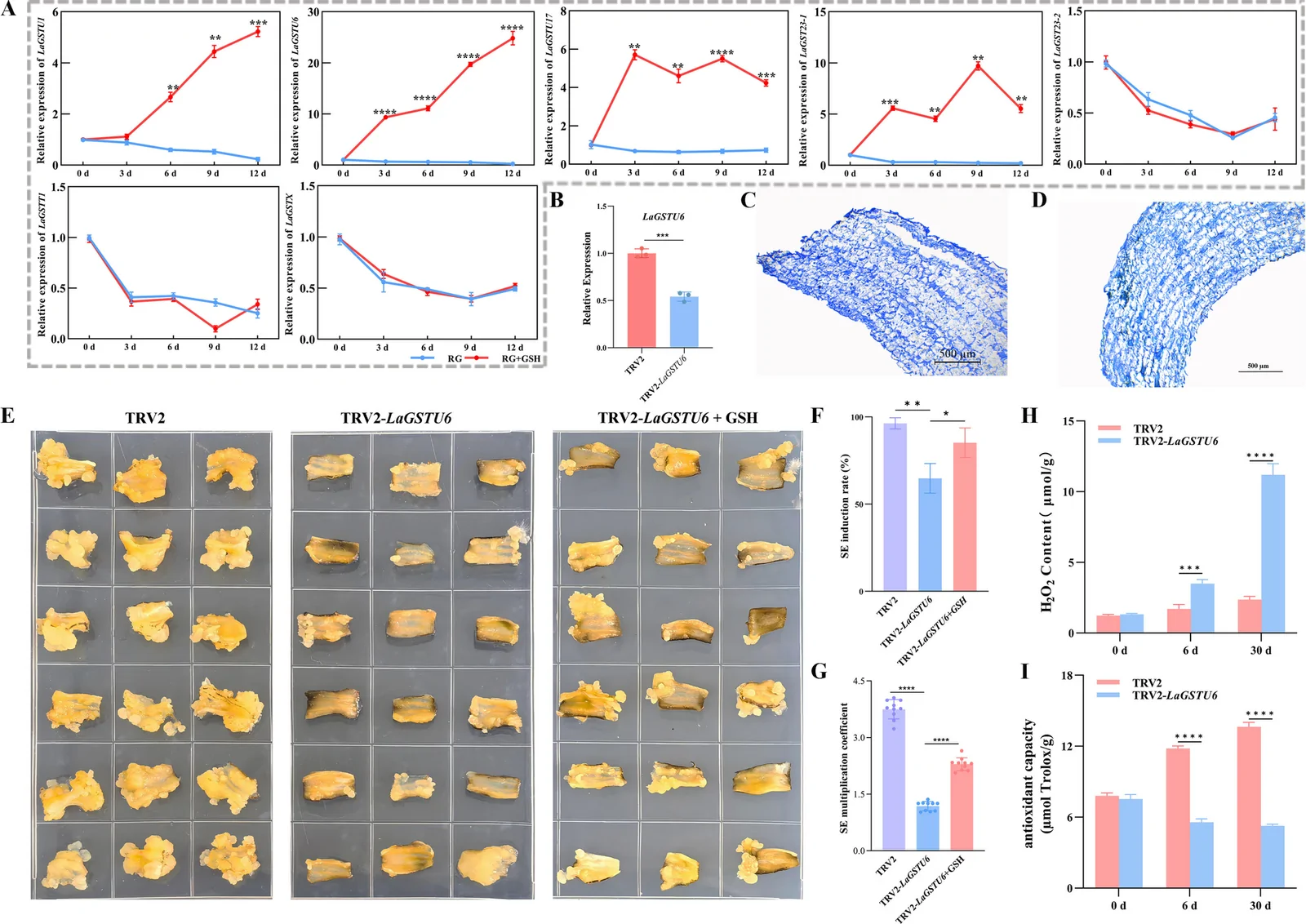
Transient silencing of *LaGSTU6* and its influence on SE in the EG. **A** Expression analysis of GST family genes in RG scales on induction media supplemented with or without GSH. **B** Expression level of *LaGSTU6* in scales with transient silencing by TRV. **C**, **D** Cell division in control (**C**) and transiently silenced (**D**) EG scales at 6 d after induction. **E**–**G** SE capacity at 30 d after induction: Phenotypic observation (**E**), induction rate (**F**), and coefficient (**G**) for control, silenced, and GSH-complemented lines. **H**, **I** Physiological indices during SE induction: H₂O₂ content (**H**) and total antioxidant capacity (**I**). Data are presented as the mean ± SD from three independent experiments (*n* = 3), each performed with three biological replicates. Statistical significance was determined via one-way ANOVA followed by Tukey’s multiple comparisons test (**, *p* < 0.01; ***, *p* < 0.001; ****, *p* < 0.0001)

WGCNA revealed that *LaGSTU6* is a hub gene within the brown module, whose expression is positively correlated with the SE capacity. Notably, six other GST family members were also enriched in this module. To investigate whether *LaGSTU6* plays a distinct central role, we systematically quantified the expression of these GST genes during early induction in the RG. The results revealed that four GST genes could be induced by GSH at this stage, but *LaGSTU6* exhibited the most sustained and significant induction response (Fig. 7A). These findings confirmed that *LaGSTU6* possesses a unique early signaling advantage within the GST family. Therefore, this gene was selected as the target for subsequent functional studies. We then transiently silenced *LaGSTU6* in the EG through virus-induced gene silencing (VIGS). Reverse transcription quantitative real-time PCR (RT-qPCR) analysis confirmed a substantial decrease in its expression 6 d after infiltration (Fig. 7B), indicating successful gene silencing. Histomorphological observation revealed that normal cell division was initiated in the control explants at 6 d after induction, whereas this critical process was notably blocked in *LaGSTU6*-silenced scales (Fig. 7C, D). Silencing also intensified tissue browning and significantly reduced the induction rate from 100% in the control to 64.81% and the induction coefficient from 3.75 to 1.23. This impairment was accompanied by a disruption of redox homeostasis, characterized by increased H₂O₂ accumulation and a concomitant decrease in the total antioxidant capacity (Fig. 7H, I). To investigate the underlying mechanism, the induction medium for the silenced explants was supplemented with GSH. This treatment resulted in a significant, although partial, rescue of SE competence: the induction rate recovered to 85.19%, and the induction coefficient increased to 2.30. However, both values remained lower than those of the control (Fig. 7E–G). In addition, the expression levels of auxin-responsive genes from the brown module were examined after the transient silencing of *LaGSTU6.* The results revealed that the expression of select auxin-responsive genes was downregulated upon *LaGSTU6* silencing, and that their expression could be partially restored by GSH supplementation (Fig. S6). Collectively, these results suggest that *LaGSTU6*, as a key component of the GSH metabolism pathway, plays an indispensable positive regulatory role in early cell fate determination during SE in lilies, likely by maintaining cellular redox balance and modulating auxin responses.

## Discussion

### Potential mechanism through which GSH induces SE in the RG

Following inoculation, dedifferentiation is initiated in plant explants under the combined influence of wounding and culture-related factors. This process is manifested as highly differentiated cells gradually losing their original structure and function, accompanied by increased anabolic metabolism, thinning of cell walls, and increased vacuolation, ultimately leading to the formation of unorganized cell clusters or callus tissue. Wounding serves as the primary trigger for dedifferentiation, inducing a cascade of physiological changes, including increased gas exchange rates, enhanced RNA synthesis (particularly satellite RNA synthesis), increased peroxidase activity, and elevated accumulation of phenolic compounds. At the molecular level, wounding-induced ROS function as signals that initiate regeneration. These ROS activate the calcium ion flux, jasmonic acid/ethylene signaling pathways, and the expression of regeneration-associated genes (e.g., *WIND1* and *ESR1*), thereby coordinating cellular reprogramming (Ma et al. 2012; Wasternack 2019; Zhu et al. 2024). However, excessive ROS accumulation in certain plants can trigger oxidative stress responses, leading to explant browning and regeneration failure. This process becomes a critical limiting factor restricting dedifferentiation and SE induction. For example, in SE-incapable *hybrid Taxodium* cell lines, multiple redox-related genes (e.g., *6PDC*, *GSTX4*, *GSTF9*, *DHAR2*, and *RER*) are expressed at low levels (Chen et al. 2024). Similarly, in this study, PIC induction disrupted the redox homeostasis in the RG, as revealed by persistent browning, reduced antioxidant capacity, and excessive H_2_O_2_ accumulation. These observations indicate that the differential ability of the EG and RG to regulate redox status during induction is a key factor influencing their SE potential.

SE essentially constitutes a stress-induced process of somatic reprogramming and totipotency activation. Classic studies in *Arabidopsis* have confirmed that stress is a direct trigger of SE. Appropriate stress treatment activates somatic reprogramming, leading to the acquisition of embryogenic competence, thereby laying a foundation for subsequent SE induction (Ikeda-Iwai et al. 2003). Further studies have confirmed that although multiple signaling pathways are involved in SE, they converge on stress signals as the central driving force. Upon exposure to exogenous stress, somatic cells experience drastic metabolic remodeling accompanied by a reorganization of the intracellular redox state, leading to the activation of numerous stress-responsive genes (Fehér, 2015). Auxin-induced oxidative stress serves as a key signal for SE in diverse plant species and plays an indispensable regulatory role during somatic embryo induction (de Moura et al. 2019; Hazubska-Przybyl et al. 2020). The synthetic auxin-type herbicide PIC has been extensively employed as an effective inducer of SE in lilies. Researchers have focused on the significant upregulation of stress-related genes during SE induction (Chen et al. 2019); however, the mechanisms underlying cellular responses to oxidative imbalance remain unclear. Our results revealed that, only under PIC treatment can the RG exhibit a significant cellular redox imbalance. Moreover, exogenous modulation to optimize the endogenous redox balance can effectively increase the SE efficiency, with a low embryogenic capacity otherwise. However, it remains unclear whether the increased embryogenic capacity in lilies results directly from the specific regulation of the GSH metabolic pathway or is mediated indirectly. Nevertheless, our findings confirm that the GSH-mediated redox balance plays an important regulatory role in driving SE induction in the RG, highlighting a key scientific question that should be investigated for elucidating the mechanisms underlying SE in *Lilium*.

Plant injury elicits concurrent regeneration and defense responses, which interact reciprocally. When this balance is skewed toward regeneration, auxin and cytokinin signaling pathways are upregulated to orchestrate tissue morphogenesis (Hernández-Coronado et al. 2022). Conversely, the predominance of defense mechanisms causes an increase in both jasmonic and salicylic acid levels, thereby inducing localized lignin deposition and potentiating wound closure. The significant accumulation of lignin precursors during RG induction supports this view. The resulting excessive lignin deposition likely causes wound closure and signal isolation, impeding auxin signal perception in the RG. Therefore, although PIC supplies an auxin-like signal, its pathway may be disrupted by this barrier or concomitant redox imbalance, explaining the failure to initiate SE with PIC alone. This inference is also consistent with the established central role of auxin in driving SE induction in lilies. Extensive studies have demonstrated that auxin is a key regulator that triggers scale dedifferentiation and SE induction, processes that are governed primarily by its signaling pathway. As demonstrated by the promotion of SE following *LpABC21* overexpression, auxin transport is crucial (Song et al. 2020). Therefore, the loss of embryogenic ability in the RG likely stems from a failure to properly activate or transduce auxin signals.

In this study, the SE capacity of the RG was successfully restored via the supplementing of PIC-containing induction medium with exogenous GSH, which significantly increased the induction rate from 0 to 33.3%. Functioning as a central redox regulator, GSH scavenges ROS through the GSH/GSSG cycle (Noctor et al. 2012) and concurrently upregulates genes involved in auxin biosynthesis (e.g., *YUC4* and *TAA1*) and signaling (e.g., *GH3.6* and *SAUR51*), which collectively facilitate SE (Kudełko and Gaj 2019; Zhang et al. 2018).

In addition to its role in driving redox homeostasis, GSH is involved in lignin degradation via Nu-class GSTs (Picart et al. 2015; Kontur et al. 2018; Marinovic et al. 2018; Rashid et al. 2024). Consistently, the results of WGCNA revealed an SE-associated module whose hub gene, i.e., *LaGSTU6*, was highly coexpressed with multiple auxin response factors (AUX/IAA and ARF), suggesting a direct transcriptional link between these pathways. Functional validation revealed that the transient silencing of *LaGSTU6* in the EG impaired SE and promoted scale browning. Both defects were partially reversed by exogenous GSH supplementation. The same silencing treatment also reduced auxin-responsive gene expression. These findings establish *LaGSTU6* as a critical integrator of GSH metabolism, redox homeostasis, and auxin signaling. The disruption of auxin responses upon *LaGSTU6* silencing results in the direct phenocopying of the regeneration defect inherent in the RG.

Thus, GSH restores SE in the RG through a *LaGSTU6*-dependent mechanism that attenuates lignification-induced physical barriers, counteracts oxidative stress, and sustains auxin signaling. Together, these actions yield cellular microenvironmental remodeling, which is conducive to reprogramming. Future studies should aim to delineate the complete regulatory network of this pathway and elucidate how *LaGSTU6* mechanistically connects to auxin signaling.

### Potential mechanisms of calcium signaling in GSH-induced SE

Calcium signaling, which constitutes a ubiquitous second messenger, is integrally involved in plant development and stress responses. During SE, calcium fluctuations activate calmodulins (CaM/CML), which in turn facilitate cell fate transitions, suggesting that calcium signaling fulfills a critical regulatory role. For instance, the Ca^2^⁺-CaM module initiates SE at the single-cell level in upland *Gossypium hirsutum* (Guo et al. 2024) and is essential for establishing the embryogenic state in *Arabidopsis* (Calabuig-Serna et al. 2023). Similarly, in lily, calmodulin expression is closely linked to the embryogenic capacity, with notable induction in the EG, indicating its essential role. In our study, WGCNA revealed high coexpression between a calmodulin gene (Cluster-85478.0) and the positive regulator *LaGSTU6* (Cluster-45914.41), indicating an interaction between calcium signaling and GSH metabolism during SE. GSH and calcium signaling are interconnected in plant physiological processes. They often function synergistically in ROS homeostasis, as observed in drought responses (Cao et al. 2023). This interplay is bidirectional: GSH-mediated redox signals can activate calcium pathways (Yu et al. 2023), while calcium signaling upregulates GSH synthesis and metabolism to increase the antioxidant capacity (Zhao et al. 2024; Zrig et al. 2021; López-Climent et al. 2014; Rentel and Knight 2004). Most directly, *MsCML10* binds and activates *MSGSTU8*, thus linking calcium sensing to the GSH-dependent network (Yu et al. 2022). The coexpression of a calmodulin with *LaGSTU6* suggests a coordinated role for calcium signaling and GSH-mediated redox homeostasis in driving the regulation of SE in lilies.

### Dynamic changes in the lignin content during SE

Lignin, which is a phenolic polymer synthesized via the phenylpropanoid pathway, is a major contributor to browning in plant tissue culture. Typically, a lower lignin content in calluses is correlated with greater embryogenic potential. However, as a crucial structural component of the cell walls, lignin influences the initiation of cell division by forming physical barriers and participating in dynamic remodeling (Kuczak and Kurczynska 2020). Consequently, lignin likely plays distinct roles at different stages of dedifferentiation and SE induction. Numerous studies have demonstrated that SE induction is accompanied by the differential expression of lignin biosynthesis genes (Chen et al. 2023) and significant fluctuations in the lignin content (Li et al. 2023). These mechanisms suggest that lignin may affect SE by regulating the degree of cell wall relaxation (Zhang et al. 2024).

In lilies, wounding-induced stress induces the sequential upregulation of ROS and jasmonic acid biosynthetic genes within 1–6 h post-wounding, followed by lignin biosynthesis gene activation (Wu et al. 2019). In this study, lignin synthesis in the dissected scale explants rapidly increased and was sustained at increased levels throughout the critical embryogenic competence acquisition period (until 6 d post-inoculation). To elucidate the functional role of lignin in shaping SE, exogenous lignin was supplemented. Surprisingly, this intervention failed to modulate the SE capacity. We attribute this observation to the following two mechanistic constraints: (1) As a macromolecular polymer, lignin requires ABC transporters (Takeuchi et al. 2018) for achieving cellular internalization and compartmentalization. Exogenously applied lignin lacks this directional transport system, thus preventing targeted accumulation. (2) Enzymatic degradation: Plant laccases oxidize exogenous lignin, thereby generating H_2_O_2_ (Perna et al. 2020). This process both inactivates lignin and exacerbates culture browning, which is consistent with the observed increase in the explant browning index. While this study did not establish a direct causal relationship between the lignin content and the acquisition of embryogenic competence, it highlighted critical limitations of exogenous lignin intervention. Future studies should aim to employ biomimetic approaches to recapitulate endogenous lignin polymerization dynamics, thereby enabling precise dissection of the cell wall remodeling mechanisms involved in SE.

## Conclusions

**Fig. 8:**
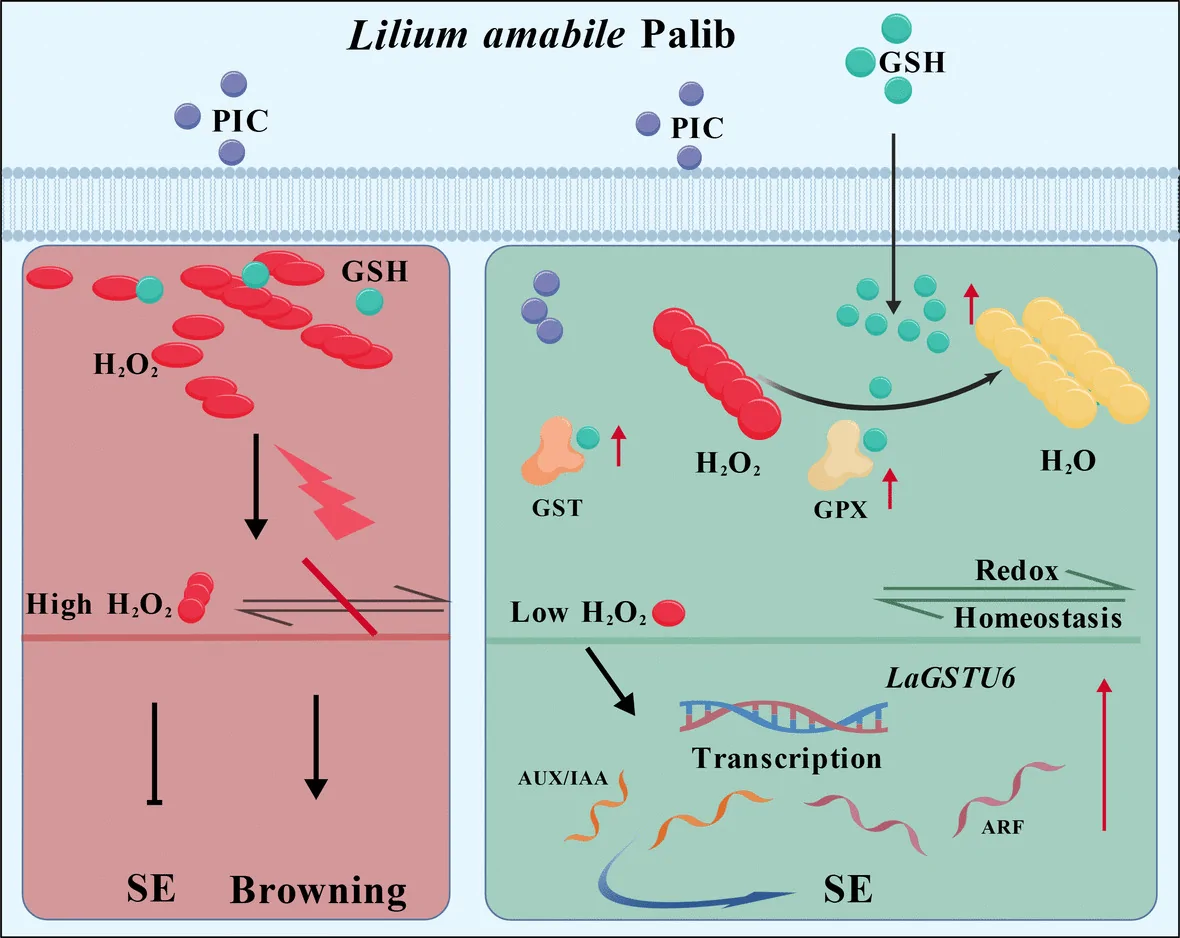
The proposed network for GSH promoted SE in *Lilium.* The network created with the assistance of GDP (agreement number: GDP2025CBBHV4) (Jiang et al. 2025b)

Our study successfully established a highly efficient SE system for *Lilium amabile* Palib. and demonstrated that modulating redox homeostasis via exogenous GSH serves as an effective strategy to overcome its genotype-dependent recalcitrance. Specifically, GSH treatment increased endogenous GSH levels and the activities of key antioxidant enzymes, including GPX and GST, which collectively contributed to the efficient scavenging of excess H₂O₂ and the subsequent alleviation of oxidative stress. Furthermore, we identified a pivotal GST family gene, namely, *LaGSTU6*. Functional characterization via transient gene silencing revealed that the silencing of *LaGSTU6* not only directly impeded the initiation of embryogenic cells, exacerbated oxidative damage, and significantly compromised the SE capacity, but also may regulate cell reprogramming by influencing the auxin response pathway, thereby establishing its crucial role in regulating SE in lilies (Fig. 8). In conclusion, our findings not only provide a robust technical platform for the genetic improvement of recalcitrant *Lilium* species but also offer novel genetic resources and theoretical insights into the redox-mediated control of plant cell totipotency.

## Materials and methods

### Plant materials

Bulblets (1.0–1.5 cm in circumference) of *Lilium martagon* var. *pilosiusculum* Freyn and *Lilium amabile* Palib., obtained after 45 days of culture under tissue culture conditions, were selected as experimental materials. The middle-layer scales were excised, trimmed at all four edges, and inoculated (concave side up) on induction medium (MS medium supplemented with 1.0 mg·L^−1^ PIC, CP8601; Coolaber, China) under complete darkness. The concentrations of GSH (CG5751; Coolaber, China) and lignin (L330880; Aladdin, China) ranged from 0 to 300.0 mg·L^−1^ and from 0 to 2.0 mg·L^−1^, respectively.

### Sample collection strategy

Scale explants from the EG and RG lines were harvested for matabolomic profiling at two critical time points, namely, preinduction (0 d) and during the early embryogenic commitment phase (6 d post-induction). Three biological replicates (*n* = 3) for each genotype collected at each time point were immediately flash-frozen in liquid nitrogen. Temporal sampling included five developmental stages (0, 3, 6, 9, and 12 d post-induction) for both genotypes. All the samples were aseptically collected, flash-frozen in liquid nitrogen, homogenized into powder, and stored at –80 ℃ in an ultralow-temperature freezer (906GP-ULTS; Thermo Fisher Scientific, USA) until RNA extraction was conducted.

### Histomorphological analysis

The conventional paraffin sectioning technique was employed for embedding. Continuous longitudinal sections (10 μm in thickness) were prepared using a rotary microtome (RM2245; Leica, Germany). The tissue sections were stained with 0.01% toluidine blue (T818873; Macklin, China) for 40 min to visualize cellular structures and then observed and photographed under a microscope (M80; Leica, Germany).

### Broadly targeted metabolomics assays and screening of DAMs

Frozen samples (50.0 mg) were homogenized in 1.2 mL of a 70.0% aqueous methanol solution (M762054; Macklin, China), vortexed and centrifuged at 12,000 rpm for 3 min to collect the supernatant. Metabolite profiling was performed using a SHIMADZU Nexera X2 UPLC system coupled with the Applied Biosystems 4500 QTRAP MS/MS platform under the chromatographic conditions described by Jiang et al. 2025a). Metabolite identification and quantification were achieved with the MetWare MWBD database (Wuhan MetWare Biotechnology Co., Ltd., China). Multivariate statistical analyses, including PCA and orthogonal partial least squares-discriminant analysis (OPLS-DA), were implemented via the prcomp package in R v4.2.1. The DAMs were screened using a threshold of |log_2_(fold change)|≥ 2.

### Detection of redox indicators in cultures

Redox-related biomarkers were quantified via the use of standardized spectrophotometric methods with assay kits (Comin Biotechnology Co., Ltd., China). H₂O₂ levels were measured through the titanium sulfate method, with the absorbance measured at a wavelength of 415 nm (H_2_O_2_−1-Y kit). The total antioxidant capacity was determined by an ABTS radical cation decolorization assay, with the absorbance measured at 734 nm (ABT-1-D kit). The GSH level was obtained via the‌ DTNB-based colorimetric method, with the absorbance measured at 412 nm (GSH-1-W kit). The levels of GSSG were measured by‌ 2-vinylpyridine derivatization followed by the DTNB reaction, with the absorbance recorded at 412 nm (GSSG-1-W kit). The glutathione reductase (GR) and GPX activities were obtained on the basis of NADPH oxidation kinetics, which were monitored by the decrease in absorbance at 412 nm (GR/GPX-1-W kit). GST activity was measured by the‌ CDNB conjugation reaction, which was monitored by the increase in absorbance at 340 nm (GST-1-W kit). All the assays were performed with three independent biological replicates, each including three technical replicates, to ensure analytical precision and statistical robustness.

### Transcriptome library construction and sequencing and screening of DEGs

Total RNA was isolated using an RN38 RNA Extraction Kit (Aidlab Biotech Co., Ltd., China) following the manufacturer’s protocols. The sequencing libraries were prepared using the Illumina Stranded mRNA Prep method detailed in previous studies (Sun et al. 2023). Unigene sequences were aligned against the KEGG, NR, Swiss-Prot, Gene Ontology (GO), Clusters of Orthologous Groups (COG)/Eukaryotic Orthologous Groups (KOG), and TrEMBL databases using DIAMOND BLASTX, and annotated accordingly. Fragments per kilobase per million mapped reads (FPKM) values were employed as measures of transcript expression levels. DEG screening was conducted using the criterion of |log_2_(fold change)|≥ 1. Multivariate analyses, including PCA, hierarchical clustering analysis, and WGCNA, were implemented in R v4.2.1 using the stats, cluster, and WGCNA packages, respectively.

### RT-qPCR analysis

Specific primers for the target gene sequences were designed using Primer 5.0. Total RNA was isolated from the samples, and cDNA was synthesized through reverse transcription with a Vazyme HiScript IV All-in-One Ultra RT SuperMix for qPCR Kit (R433, Vazyme, China). RT-qPCR was performed on an ABI 7500 Real-Time PCR System (Life Technologies, CA, USA) with the use of SYBR qPCR Master Mix (Q711, Vazyme, China). Relative gene expression levels were normalized to the expression level of the internal reference gene *Actin* and calculated via the 2^−ΔΔCt^ method. All experiments included three biological replicates and three technical replicates per sample. The primer sequences are detailed in Table S1.

### Virus-induced transient silencing of *LaGSTU6*

The *LaGSTU6* gene was transiently silenced in *Lilium martagon* var. *pilosiusculum* Freyn scales by VIGS. Vector construction and preparation of the *Agrobacterium* infiltration suspension followed the method described by Fan et al. (2023), while the infiltration procedure was adapted from the tissue culture system of Pan et al. (2023) with modifications for scale explants. The TRV1, TRV2 (empty vector), and TRV2-*LaGSTU6* constructs were individually transformed into *Agrobacterium* strain GV3101. After resuspension in infiltration buffer (10 mM MES, 10 mM MgCl₂, and 200 μM acetosyringone, pH 5.6) to an OD₆₀₀ = 0.6 and incubation in the dark for 3 h, the TRV1 suspension was mixed separately with equal volumes of TRV2 or TRV2-*LaGSTU6* suspensions to prepare coinfiltration working solutions. The middle layers of the scales were excised on all four sides and, together with 15 mL of the working solution, placed in a 50-mL syringe for vacuum infiltration. The plunger was gently drawn to the 40-mL mark, held for 1 min, and then slowly released. The scales were subsequently blotted dry on sterile filter paper and inoculated on MS media supplemented with 1.0 mg·L^−1^ PIC and 200 μM acetosyringone for SE induction in the dark. Two experimental groups were established, namely, an empty vector control (TRV1 + TRV2) group and a *LaGSTU6*-silencing group (TRV1 + TRV2-*LaGSTU6*). In each group, a total of 90 scale explants were used and divided into three independent biological replicates (30 scales per replicate). All steps were performed under aseptic conditions.

### Statistical analyses

Statistical analyses were performed in GraphPad Prism 9.5.1. Between-group comparisons for any two experimental conditions were assessed using Student’s *t* test, with statistical significance set to* p* < 0.05. For comparisons across three or more groups, analysis of variance (ANOVA) was applied, followed by appropriate post hoc tests as applicable, thereby using the same significance threshold.

## Supplementary Information

Supplementary Material 1: Fig. S1. Metabolomic analysis. Fig. S2. Effects of lignin on the somatic embryogenic capacity of EG. Fig. S3. ‌KEGG enrichment analysis of DEGs. Fig. S4. Validation of DEGs by RT-qPCR. Fig. S5. ‌KEGG enrichment analysis of DAMs and DEGs. Fig. S6. Quantitative analysis of genes responsive to auxin.

Supplementary Material 2: Table S1. RT-qPCR primers. Table S2. Effects of the species and concentration of plant growth regulators and types of explants on SE induction of Lilium amabile Palib. Table S3. Summary of RNA-seq data. Table S4. Statistics of the assembly data. Table S5. Overview of the functional annotations of unigenes.

Supplementary Material 3: Table S6. Intergenic correlations and functional annotations within the brown module.

## Data Availability

Data will be available from the corresponding author upon reasonable request.

## Abbreviations

ANOVA: One-way analysis of variance
COG: Clusters of orthologous groups
DAMs: Differentially accumulated metabolites
DEGs: Differentially expressed genes
EG: Efficient genotype
FPKM: Fragments per kilobase per million mapped fragments
GO: Gene ontology
GPX: Glutathione peroxidase
GR: Glutathione reductase
GSH: Glutathione
GSSG: Oxidized glutathione
GST: Glutathione S-transferase
H₂O₂: Hydrogen peroxide
KEGG: Kyoto Encyclopedia of Genes and Genomes
KOG: Eukaryotic orthologous groups
OPLS-DA: Orthogonal partial least squares-discriminant analysis
PCA: Principal component analysis
PIC: Picloram
PSK: Phytosulfokine
RT-qPCR: Reverse transcription quantitative real-time PCR
RG: Recalcitrant genotype
RNA-seq: RNA sequencing
ROS: Reactive oxygen species
SD: Standard deviation
SE: Somatic embryogenesis
VIGS: Virus induced gene silencing
WGCNA: Weighted gene coexpression network analysis
