# Supplementary material for: The glutathione S-transferase gene LaGSTU6 promotes somatic embryogenesis in a recalcitrant lily genotype by regulating scales redox homeostasis
Source: Mol Hortic. 2026 Oct 9;6:71. doi: 10.1186/s43897-026-00244-9 (PMC13651384; doi:10.1186/s43897-026-00244-9)
Supplement: Supplementary file 1 [file 43897_2026_244_MOESM1_ESM.docx]

**Fig. S1** Metabolomic analysis.

**A** PCA score plot of detected metabolites from samples. **B**‌ Distribution of metabolite categories.

**Fig. S2** Effects of lignin on the SE capacity of EG.

**A** MS + 1.0 mg·L^-1^ PIC. **B** MS + 1.0 mg·L^-1^ PIC + 0.5 mg·L^-1^ lignin. **C** MS + 1.0 mg·L^-1^ PIC + 1.0 mg·L^-1^ lignin. **D** MS + 1.0 mg·L^-1^ PIC + 2.0 mg·L^-1^ lignin.

**Fig. S3 ‌**KEGG enrichment analysis of DEGs.

**A** KEGG enrichment analysis of DEGs in the EG0d VS EG6d comparison. **B** KEGG enrichment analysis of DEGs in the RG0d VS RG6d comparison. **C** KEGG enrichment analysis of DEGs in the EG6d VS RG6d cross-group comparison.

**Fig. S4** Validation of DEGs by RT-qPCR.‌ **‌A** DEGs of EG. **B** DEGs of RG.

**Fig. S5** ‌KEGG enrichment analysis of DAMs and DEGs.

**A** KEGG enrichment analysis of DAMs and DEGs in the EG0d VS EG6d comparison. **B** KEGG enrichment analysis of DAMs and DEGs in the RG0d VS RG6d comparison.

**Fig. S6** Quantitative analysis of genes responsive to auxin.

Data are presented as the mean ± SD from three independent experiments, each performed with three biological replicates (n = 3). Statistical significance was determined by one‑way ANOVA followed by Tukey’s multiple comparisons test (**, *p* < 0.01; ***, *p* < 0.001; ****, *p* < 0.0001). No notation indicates no significant difference.
