# Supplementary material for: The glutathione S-transferase gene LaGSTU6 promotes somatic embryogenesis in a recalcitrant lily genotype by regulating scales redox homeostasis
Source: Mol Hortic. 2026 Oct 9;6:71. doi: 10.1186/s43897-026-00244-9 (PMC13651384; doi:10.1186/s43897-026-00244-9)
Supplement: Supplementary file 2 [file 43897_2026_244_MOESM2_ESM.docx]

**Table S1** RT-qPCR primers.

| Primer name | Primer sequence (5'-3') |
| --- | --- |
| *Action*-F | ACGACTACTGCTGAACGAGAAA |
| *Action*-R | AAGGAAGGCTGGAAGAGGAC |
| Cluster-38303.32/*LaGSTU1*-q-F | CGGGGCTGACATTCTCCAAC |
| Cluster-38303.32/*LaGSTU1*-q-R | GCATCGCCAACAATAGCTCGA |
| Cluster-45914.41/*LaGSTU6*-q-F | TTGTGATGCGAGCAAGGGTT |
| Cluster-45914.41/*LaGSTU6*-q-R | TTTGTAGACGGGATTAGATTTCAGC |
| Cluster-48395.22/*LaGSTU17*-q-F | ATCCTCCCCAGTGATGCTTATG |
| Cluster-48395.22/*LaGSTU17*-q-R | CTCAGTCGCTGCCTCCCTATT |
| Cluster-51698.0/*LaGST23-1*-q-F | TGCTACCCCATCATGCCTAAA |
| Cluster-51698.0/*LaGST23-1*-q-R | CACTGCCTTCTGTTGCTCTTCTT |
| Cluster-51698.4/*LaGST23-2*-q-F | TCATGCCTAAAGCTCCCTACCA |
| Cluster-51698.4/*LaGST23-2*-q-R | AAGGAATCGCCACCGAAGAA |
| Cluster-86481.1/*LaGSTT1*-q-F | GTGCTTTTCCAGGAGTCCCAGAT |
| Cluster-86481.1/*LaGSTT1*-q-R | CAAGTGCGGGTGCTACAACAA |
| Cluster-38303.21/*LaGSTX*-q-F | GGCAAGAGGCTGCGAAGAA |
| Cluster-38303.21/*LaGSTX-*q-R | ACATCAACGAACCCGAAAACAT |
| Cluster-38300.3-q-F | CAGGAGACGGTCCACTTCGA |
| Cluster-38300.3-q-R | CTGGTGGTGACGCCCTTGTA |
| Cluster-52503.5-q-F | GGGCTGACATTCTCCAACGA |
| Cluster-52503.5-q-R | CGCACCATTCATCCCCACAA |
| Cluster-85478.0-q-F | ACCGATCTAGCCGCCATGA |
| Cluster-85478.0-q-R | TCGTCCCGCTCGTCCATTA |
| Cluster-97767.0-q-F | GAACTTGTCACAAACGGGGC |
| Cluster-97767.0-q-R | TGGACTGTCTCGTCTTTCGC |
| Cluster-96544.2-q-F | TGAGGCGCATGAAGTTGGAT |
| Cluster-96544.2-q-R | AGATTGTTCTGCGCCCATGA |
| Cluster-34164.2-q-F | TGGCGTAACTCACCTGTCAC |
| Cluster-34164.2-q-R | CGTTTCTGGTTGGGAGAGCT |
| Cluster-8489.21-q-F | ATAAGGTCTTTGGGGCCAGC |
| Cluster-8489.21-q-R | GGGCCTCATACGAGATGGTG |
| Cluster-8489.18-q-F | CACCATCTCGTATGAGGCCC |
| Cluster-8489.18-q-R | CCATTGACAAGACCACGGGA |
| Cluster-67390.1-q-F | CAGCTTCGACACATTGCTCG |
| Cluster-67390.1-q-R | CTCGCTCCGTACTTCGACT |
| Cluster-55751.8-q-F | TGGGAATGGATGGTGCAGTC |
| Cluster-55751.8-q-R | CAGTCCCTGGTGCATTTCCT |
| Cluster-55748.27-q-F | TCCCCATTCATGACCTTGGC |
| Cluster-55748.27-q-R | CGACACAACCATGGACAGGA |
| Cluster-55748.26-q-F | TCCCCATTCATGACCTTGGC |
| Cluster-55748.26-q-R | CGACACAACCATGGACAGGA |
| Cluster-94152.3/*LaARF19-2*-q-F | ACGGCTGTAGCAATGAACCA |
| Cluster-94152.3/*LaARF19-2*-q-R | GGTGGTTGCTGCTGAAGTTG |
| Cluster-94152.0/*LaARF19-1*-q-F | ACGGCTGTAGCAATGAACCA |
| Cluster-94152.0/*LaARF19-1*-q-R | GGTGGTTGCTGCTGAAGTTG |
| Cluster-90573.2/*LaARF15-2*-q-F | GTTGTGGACGTCAGGCTACA |
| Cluster-90573.2/*LaARF15-2*-q-R | CCCTCCTCTTCTCGCGTTTT |
| Cluster-90573.0/*LaARF15-1*-q-F | CGCAGCACGAAAGTTCACAA |
| Cluster-90573.0/*LaARF15-1*-q-R | GGTCATTCAGAAGCCCCTCC |
| Cluster-56757.0/*LaARF9*-q-F | ACTTCCCTCAAGGCCACATG |
| Cluster-56757.0/*LaARF9*-q-R | GAGGATCTTGGAGGGGAGGT |
| Cluster-19209.3/*LaIAA13*-q-F | TAGTGAAGGAGGAGCGAGTGG |
| Cluster-19209.3/*LaIAA13-*q-R | GGAGATGATCGTGACCGAATAG |
| Cluster-51407.0-q-F | GCTGTCACTGTGGTTCTCGA |
| Cluster-51407.0-q-R | TTCGCCAAGCCCATCATCTT |
| *LaIAA1*-q-F | CTTATGAGGACAAGGATGGAGACT |
| *LaIAA1*-q-R | TCCCGGAACTAGATTGACAGC |
| *LaIAA1-1*-q-F | GTCCGTGGAGACAGCGAAGA |
| *LaIAA1-1*-q-R | CGACTAACCGATTGGCAGCAT |
| *LaIAA1-2*-q-F | GATAAGGATGGCGATTGGATG |
| *LaIAA1-2*-q-R | ACCTAAATTGACGGCCTCTGA |
| *LaIAA10*-q-F | GCGAGTGGATGTGGCAGAA |
| *LaIAA10*-q-R | GGAGATGATCGTGACCGAATAGA |
| *LaIAA30*-q-F | AGAGGAGAAGCATAGCAACAACA |
| *LaIAA30*-q-R | TCCCTTCCATTGACCCCAT |
| *LaIAA33*-q-F | GTACCAGGGTACTTGGTTGC |
| *LaIAA33*-q-R | CCATCACATTTAGTTTCCGCTCT |

**Table S2** Effects of the species and concentration of plant growth regulators and types of explants on SE induction of *Lilium amabile* Palib.

| Types of explants | Treatment | Induce the 30 d state description | SE induction rate（%） |
| --- | --- | --- | --- |
| Scale | MS | Adventitious buds developed on 33.3% of the scales, while the remainder exhibited browning symptoms. | 0.0 |
|  | MS + 0.5 mg·L^-1^ PIC | 100.0% of scales exhibited progressive browning followed by complete necrosis. | 0.0 |
|  | MS + 1.0 mg·L^-1^ PIC | 100.0% of scales exhibited progressive browning followed by complete necrosis. | 0.0 |
|  | MS + 2.0 mg·L^-1^ PIC | 100.0% of scales exhibited progressive browning followed by complete necrosis. | 0.0 |
|  | MS + 0.5 mg·L^-1^ NAA + 0.5 mg·L^-1^ 6-BA | Adventitious buds developed on 63.3% of the scales, the remainder exhibited browning symptoms. | 0.0 |
|  | MS + 0.5 mg·L^-1^ NAA + 1.0 mg·L^-1^ 6-BA | Adventitious buds developed on 60.0% of the scales, the remainder exhibited browning symptoms. | 0.0 |
|  | MS + 0.5 mg·L^-1^ NAA + 1.5 mg·L^-1^ 6-BA | Adventitious buds developed on 13.3% of the scales, the remainder exhibited browning symptoms. | 0.0 |
|  | MS +0.5 mg·L^-1^ NAA + 0.5 mg·L^-1^ TDZ | Adventitious buds developed on 13.3% of the scales, the remainder exhibited browning symptoms. | 0.0 |
|  | MS +0.5 mg·L^-1^ NAA + 1.0 mg·L^-1^ TDZ | Dysplastic buds developed on 10.0% of the scales, the remainder exhibited browning symptoms. | 0.0 |
|  | MS +0.5 mg·L^-1^ NAA + 1.5 mg·L^-1^ TDZ | Dysplastic buds developed on 46.7% of the scales, the remainder exhibited browning symptoms. | 0.0 |
|  | MS +0.5 mg·L^-1^ NAA + 0.5 mg·L^-1^ KT | Adventitious buds developed on 43.3% of the scales, the remainder exhibited browning symptoms. | 0.0 |
|  | MS +0.5 mg·L^-1^ NAA + 1.0 mg·L^-1^ KT | Adventitious buds developed on 26.7% of the scales, the remainder exhibited browning symptoms. | 0.0 |
|  | MS +0.5 mg·L^-1^ NAA + 1.5 mg·L^-1^ KT | Adventitious buds developed on 30.0% of the scales, the remainder exhibited browning symptoms. | 0.0 |
|  | MS +0.5 mg·L^-1^ 2,4-D + 0.5 mg·L^-1^ 6-BA | Adventitious buds developed on 23.3% of the scales, the remainder exhibited browning symptoms. | 0.0 |
|  | MS +0.5 mg·L^-1^ 2,4-D + 1.0 mg·L^-1^ 6-BA | Adventitious buds developed on 30.0% of the scales, the remainder exhibited browning symptoms. | 0.0 |
|  | MS +0.5 mg·L^-1^ 2,4-D + 1.5 mg·L^-1^ 6-BA | Adventitious buds developed on 13.3% of the scales, the remainder exhibited browning symptoms. | 0.0 |
|  | MS +0.5 mg·L^-1^ 2,4-D + 0.5 mg·L^-1^ TDZ | Dysplastic buds developed on 6.7% of the scales, the remainder exhibited browning symptoms. | 0.0 |
|  | MS +0.5 mg·L^-1^ 2,4-D + 1.0 mg·L^-1^ TDZ | Dysplastic buds developed on 6.7% of the scales, the remainder exhibited browning symptoms. | 0.0 |
|  | MS +0.5 mg·L^-1^ 2,4-D + 1.5 mg·L^-1^ TDZ | 100.0% of scales exhibited progressive browning followed by complete necrosis. | 0.0 |
|  | MS +0.5 mg·L^-1^ 2,4-D + 0.5 mg·L^-1^ KT | Adventitious buds developed on 40.0% of the scales, the remainder exhibited browning symptoms. | 0.0 |
|  | MS +0.5 mg·L^-1^ 2,4-D + 1.0 mg·L^-1^ KT | Adventitious buds developed on 43.3% of the scales, the remainder exhibited browning symptoms. | 0.0 |
|  | MS +0.5 mg·L^-1^ 2,4-D + 1.5 mg·L^-1^ KT | Adventitious buds developed on 46.7% of the scales, the remainder exhibited browning symptoms. | 0.0 |
| Leaf | MS | 100.0% of leaves exhibited progressive browning followed by complete necrosis. | 0.0 |
|  | MS + 0.5 mg·L^-1^ PIC | 100.0% of leaves exhibited progressive browning followed by complete necrosis. | 0.0 |
|  | MS + 1.0 mg·L^-1^ PIC | 100.0% of leaves exhibited progressive browning followed by complete necrosis. | 0.0 |
|  | MS + 2.0 mg·L^-1^ PIC | 100.0% of leaves exhibited progressive browning followed by complete necrosis. | 0.0 |
| Root | MS | The length of the root increased. | 0.0 |
|  | MS + 0.5 mg·L^-1^ PIC | The length of the root increased. | 0.0 |
|  | MS + 1.0 mg·L^-1^ PIC | Non-embryonic callus formation was observed in 33.3% of root explants**.** | 0.0 |
|  | MS + 2.0 mg·L^-1^ PIC | Non-embryonic callus formation was observed in 40.0% of root explants. | 0.0 |

**Table S3** Summary of RNA-seq data.

| Sample | Raw Reads | Clean Reads | Clean Base（G） | Q20(%) | Q30(%) | GC Content (%) |
| --- | --- | --- | --- | --- | --- | --- |
| EG0d1 | 46585788 | 45717248 | 6.86 | 97.56 | 93.25 | 48.82 |
| EG0d2 | 46757232 | 45727674 | 6.86 | 97.24 | 92.46 | 48.75 |
| EG0d3 | 48003796 | 46997444 | 7.05 | 97.54 | 93.12 | 48.48 |
| EG3d1 | 47822614 | 46954106 | 7.04 | 97.40 | 92.91 | 49.45 |
| EG3d2 | 46670192 | 45632118 | 6.84 | 97.44 | 92.98 | 48.12 |
| EG3d3 | 47248968 | 45886302 | 6.88 | 97.29 | 92.66 | 49.30 |
| EG6d1 | 46274430 | 44363842 | 6.65 | 97.45 | 93.00 | 48.51 |
| EG6d2 | 49270058 | 47992604 | 7.20 | 97.61 | 93.41 | 49.63 |
| EG6d3 | 45938744 | 43579152 | 6.54 | 97.57 | 93.26 | 48.08 |
| EG9d1 | 47669584 | 45829010 | 6.87 | 97.78 | 93.84 | 49.29 |
| EG9d2 | 44911622 | 43859424 | 6.58 | 97.33 | 92.84 | 49.96 |
| EG9d3 | 47706266 | 46559410 | 6.98 | 97.36 | 92.84 | 49.14 |
| EG12d1 | 48427572 | 47231288 | 7.08 | 97.35 | 92.81 | 50.07 |
| EG12d2 | 46587156 | 45582550 | 6.84 | 97.34 | 92.84 | 49.85 |
| EG12d3 | 49143306 | 48123242 | 7.22 | 97.36 | 92.81 | 49.36 |
| RG0d1 | 46423060 | 45360086 | 6.80 | 97.32 | 92.73 | 49.22 |
| RG0d2 | 46547440 | 45449058 | 6.82 | 97.71 | 93.61 | 48.96 |
| RG0d3 | 48739270 | 47623810 | 7.14 | 97.38 | 92.85 | 49.28 |
| RG3d1 | 46264100 | 45177130 | 6.78 | 97.45 | 93.06 | 49.80 |
| RG3d2 | 48093714 | 47268572 | 7.09 | 97.32 | 92.73 | 49.91 |
| RG3d3 | 47416370 | 46266732 | 6.94 | 97.39 | 92.87 | 50.05 |
| RG6d1 | 47361844 | 46301966 | 6.95 | 97.19 | 92.41 | 50.45 |
| RG6d2 | 49173586 | 48243148 | 7.24 | 97.47 | 93.03 | 50.59 |
| RG6d3 | 48728124 | 47514078 | 7.13 | 97.54 | 93.15 | 50.45 |
| RG9d1 | 46257528 | 45567636 | 6.84 | 97.59 | 93.33 | 50.62 |
| RG9d2 | 4800934 | 46750572 | 7.01 | 97.49 | 93.08 | 50.83 |
| RG9d3 | 44711884 | 44024800 | 6.60 | 97.49 | 93.06 | 50.76 |
| RG12d1 | 53411548 | 52140504 | 7.82 | 97.47 | 93.09 | 50.85 |
| RG12d2 | 47325004 | 46316040 | 6.95 | 97.54 | 93.20 | 50.97 |
| RG12d3 | 46954682 | 45850122 | 6.88 | 97.49 | 93.07 | 50.96 |

**Table S4** Statistics of the assembly data.

| Type | Number | Mean length | N50 | N90 | Total bases |
| --- | --- | --- | --- | --- | --- |
| Transcript | 365,389 | 779 | 1,257 | 306 | 284,504,053 |
| Unigene | 198,905 | 1,038 | 1,461 | 472 | 206,463,980 |

**Table S5** Overview of the functional annotations of unigenes.

| Database | Number of genes | Percentage (%) |
| --- | --- | --- |
| KEGG | 81,919 | 41.18 |
| Nr | 109,576 | 55.08 |
| SwissProt | 81,795 | 41.11 |
| TrEMBL | 110,820 | 55.70 |
| KOG | 63,973 | 32.16 |
| GO | 950,02 | 47.75 |
| Pfam | 74,297 | 37.34 |
| Annotated in at least one Database | 115,595 | 58.10 |
| Total Unigenes | 198,950 | 100.00 |
